# A Giant Mature Cystic Teratoma Mimicking a Pleural Effusion

**DOI:** 10.1155/2016/1259175

**Published:** 2016-01-28

**Authors:** Mustafa Erman Dorterler, Mehmet Emin Boleken, Sezen Koçarslan

**Affiliations:** ^1^Department of Pediatric Surgery, Harran University, Şanlıurfa, Turkey; ^2^Department of Pathology, Harran University, Şanlıurfa, Turkey

## Abstract

The vast majority of teratomas originating from more than a single germ layer are benign. Often, such teratomas are initially asymptomatic. Later symptoms are caused by the weight per se of the teratoma and include chest pain, cough, dyspnea, and/or recurrent attacks of pneumonia. A mediastinal teratoma is treated by total surgical resection of the mass. Here, we report a case of giant mature cystic teratoma mimicking a pleural effusion in the thorax at the 7-month-old female patient with a symptom of persistent pulmonary infection and tachypnea.

## 1. Introduction

Although benign teratomas are often located in the anterior of the mediastinum, they may also develop within the parenchyma or in paravertebral areas. Such teratomas are often asymptomatic. The symptoms that later arise are caused by the weight per se of the teratoma and include chest pain, cough, dyspnea, and/or recurrent attacks of pneumonia. The tumor may adhere to surrounding structures and resection of the pericardium, pleura, and/or lungs may be necessary [1].

## 2. Presentation of Case

**Figure 1:**
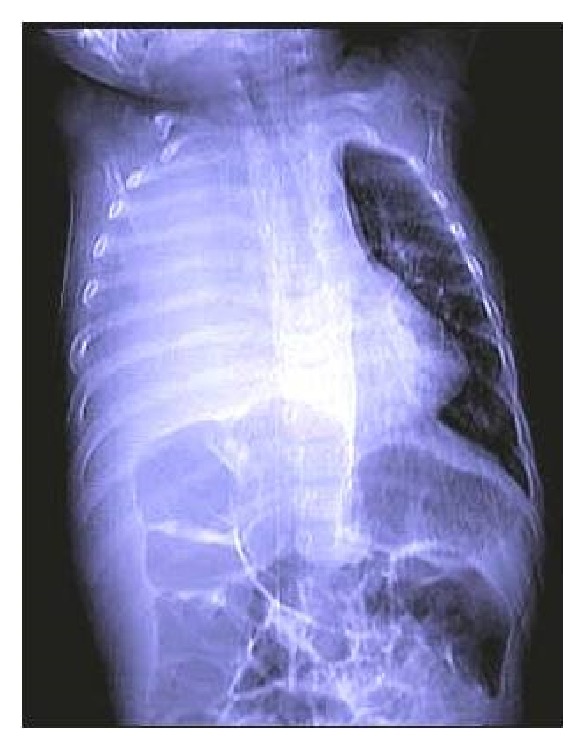
A preoperative PA radiograph.

A 7-month-old female patient was referred to our clinic because of a persistent pulmonary infection and shortness of breath that was exacerbated at rest. We suspected a pleural effusion in the right hemithorax but tube thoracostomy did not afford any improvement (Figure 1). We then learnt that the patient had received 20-day courses of various antibiotics. As the patient was tachypneic and cyanotic on physical examination, we placed her on nasally delivered oxygen. Upon auscultation, we found that the right hemithorax did not participate in respiration. A pulmonary radiograph revealed that an opacity completely filled the right mediastinum.

**Figure 2:**
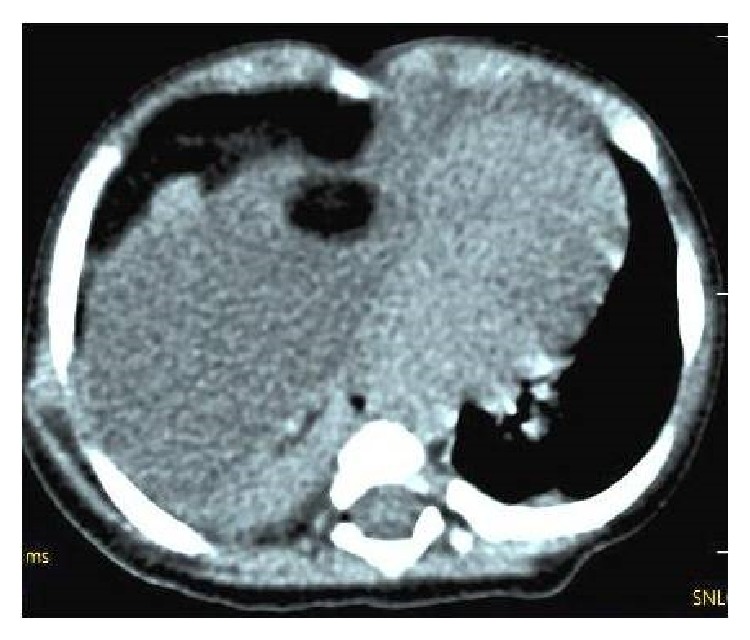
A CT image of the thorax.

**Figure 3:**
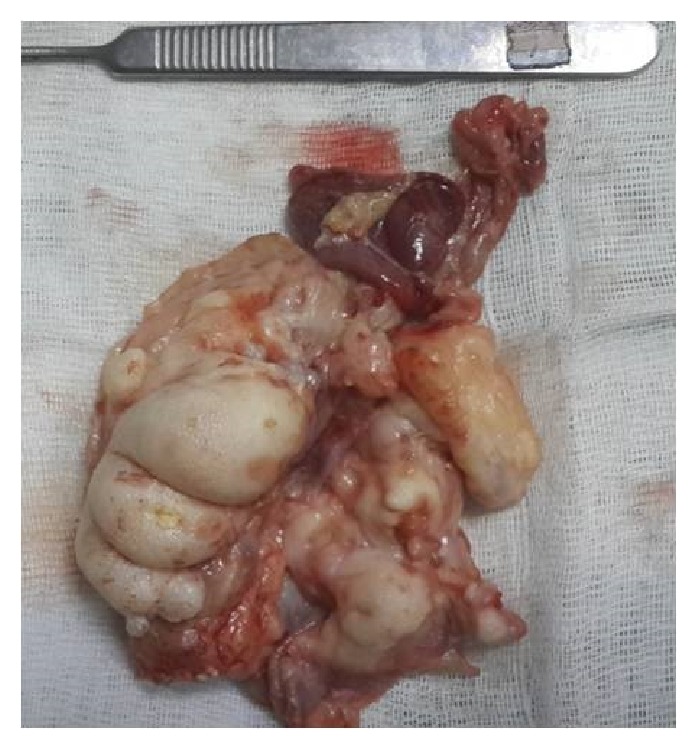
Macroscopic appearance of the excised teratoma.

**Figure 4:**
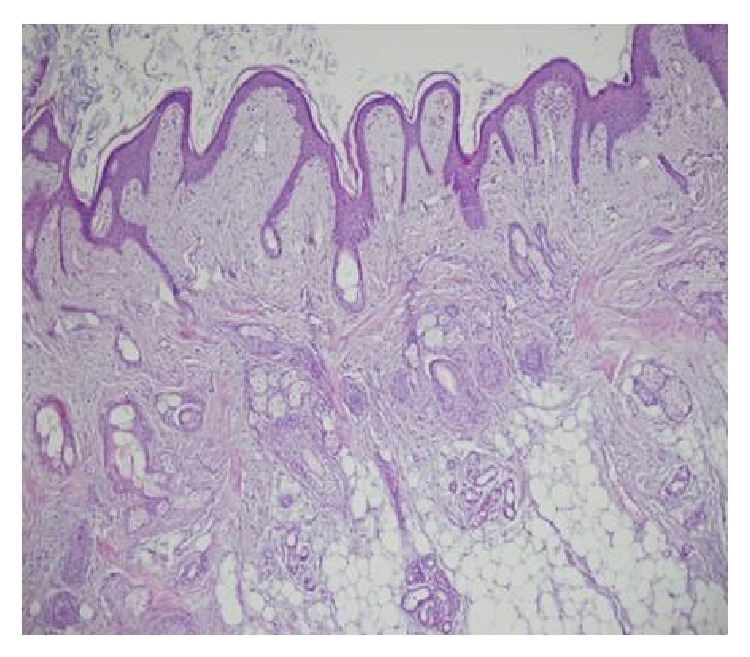
Histopathological features of the teratoma.

Routine hematological test results and abdominal sonographic findings were within normal limits. The Mantoux test was negative. The levels of tumor markers (*α*-fetoprotein and *β*-hCG) were within normal limits. On CT examination, a large cyst was seen to have invaded the right hemithorax accompanied by shifting of the mediastinum to the left (Figure 2). Our initial diagnosis was a benign teratoma evident both clinically and radiologically, and we performed a right posterolateral between 4 and 5 interthoracal thoracotomy for both diagnostic and treatment purposes. The tumor completely filled the right hemithorax and adhered extensively to the mediastinal pleura and pericardium. The tumor was totally removed (Figure 3). On histopathological examination, the tumor was seen to be a benign mature teratoma containing layers of smooth muscle, respiratory epithelium, mature fatty tissue, cartilage, and connective tissue (Figure 4). At the postoperative 3-month follow-up, no abnormality was evident.

## 3. Discussion

The vast majority of teratomas originating from more than a single germ layer are benign. Teratomas may develop in both undifferentiated embryonal tissues and fully differentiated tissues such as the skin and associated structures, teeth, nerve bundles, ganglion cells, and the brain and intestine [2]. Tumors have been noted in all age groups but develop predominantly in young adults [3]. Unusually, our present patient was aged only 7 months.

About 97% of all mediastinal lesions can be diagnosed by direct thoracic radiography. Radiologically, the mass is usually regularly contoured. Tomography can define the size of the lesion, identify tissues within the teratoma, and reveal where the tumor compresses surrounding tissue [3–6]. Unusually, direct pulmonary radiography of our present case yielded a pattern mimicking that of a pleural effusion. Computed tomography was required for diagnosis.

Depending on the location of a mediastinal mass, surgery can afford both a definitive diagnosis and curative resection. However, if total resection is impossible because the mass adheres densely to adjacent structures (the pericardium, major vascular structures, hilar structures, or the esophagus), subtotal resection may be possible and is associated with good prognosis [7]. Preoperative diagnosis of mediastinal masses is very difficult. Surgery is essential for both diagnostic and treatment (total removal) purposes [8].

In our current case, the mass adhered widely to the pleura and pericardium. We carefully freed all tissues around the mass and achieved total resection. No recurrence was evident 3 months later.

## 4. Conclusion

The thoracal site remains a rare site for teratoma occurrence in children with tachypnea and persistent pulmonary infection as an ever-present danger. Early diagnosis is crucial, allowing for early recognition and intervention. Careful and meticulous complete extirpation is the goal of treatment. If the benign teratoma is totally resected, this will provide an adequate cure without the need for chemotherapy or radiotherapy. We resected a giant mature mediastinal teratoma, which occupied the entire right hemithorax and compressed the right lung and in 7-month-old girl.
